# Sodium oxybate in treatment-resistant rapid-eye-movement sleep behavior disorder

**DOI:** 10.1093/sleep/zsad103

**Published:** 2023-04-13

**Authors:** Emmanuel H During, Beatriz Hernandez, Mitchell G Miglis, Oliver Sum-Ping, Anahid Hekmat, Ana Cahuas, Adrian Ekelmans, Fuyumi Yoshino, Emmanuel Mignot, Clete A Kushida

**Affiliations:** Department of Psychiatry and Behavioral Sciences, Stanford University, Palo Alto, CA, USA; Department of Neurology and Neurological Sciences, Stanford University, Palo Alto, CA, USA; Department of Neurology, Division of Movement Disorders, Icahn School of Medicine at Mount Sinai, New York, NY, USA; Department of Psychiatry and Behavioral Sciences, Stanford University, Palo Alto, CA, USA; Sierra Pacific Mental Illness Research Education and Clinical Center (MIRECC), Veterans Affairs Palo Alto Health Care System, Palo Alto, CA, USA; Department of Psychiatry and Behavioral Sciences, Stanford University, Palo Alto, CA, USA; Department of Neurology and Neurological Sciences, Stanford University, Palo Alto, CA, USA; Department of Psychiatry and Behavioral Sciences, Stanford University, Palo Alto, CA, USA; Department of Psychiatry and Behavioral Sciences, Stanford University, Palo Alto, CA, USA; Department of Psychiatry and Behavioral Sciences, Stanford University, Palo Alto, CA, USA; Department of Psychiatry and Behavioral Sciences, Stanford University, Palo Alto, CA, USA; Department of Psychiatry and Behavioral Sciences, Stanford University, Palo Alto, CA, USA; Department of Medicine, Osaka University, Osaka, Japan; Department of Psychiatry and Behavioral Sciences, Stanford University, Palo Alto, CA, USA; Department of Psychiatry and Behavioral Sciences, Stanford University, Palo Alto, CA, USA

**Keywords:** REM sleep behavior disorder, Parkinson’s disease, sodium oxybate, parasomnia

## Abstract

**Study Objectives:**

Symptomatic therapies for rapid-eye-movement (REM) sleep behavior disorder (RBD) are limited. Sodium oxybate (SXB), a gamma-aminobutyric acid (GABA)-B agonist, could be effective but has not been evaluated against placebo.

**Methods:**

This double-blind, parallel-group, randomized, placebo-controlled trial in 24 participants was conducted at the Stanford Sleep Center. Patients were adults with definite iRBD or Parkinson’s disease and probable RBD (PD-RBD), and persistence of ≥ 2 weekly episodes despite standard therapy. Patients were randomized 1:1 to receive SXB during a 4-week titration followed by a 4-week stable dosing period. Primary outcome was number of monthly RBD episodes according to a diary filled by patients and partners. Secondary outcomes were severity, number of severe RBD episodes, and objective RBD activity on video polysomnography.

**Results:**

Twelve iRBD and 12 PD-RBD participated (mean 65.8 years), and 22 (*n* = 10 SXB, 12 placebo) completed the study. Although no significant between-group difference was found, SXB showed reduction of monthly RBD episodes by 23.1 (95% CI −36.0, −10.2; *p* = 0.001) versus 10.5 with placebo (95% CI, −22.6, 1.6; *p* = 0.087). Improvement from baseline was similarly observed for RBD overall severity burden (each episode weighted for severity), number of severe episodes, and objective RBD activity per video-polysomnography. Two participants receiving SXB withdrew due to anxiety and dizziness. The majority of adverse events are otherwise resolved with dose adjustment.

**Conclusion:**

SXB could reduce RBD symptoms; however, response was inconsistent and a large placebo effect was observed across patient-reported outcomes. Larger studies using objective endpoints are needed.

**Clinical Trial:**

Treatment of REM Sleep Behavior Disorder (RBD) With Sodium Oxybate

https://clinicaltrials.gov/ct2/show/NCT04006925 ClinicalTrials.gov identifier: NCT04006925

## Graphical abstract

**Graphical Abstract:**
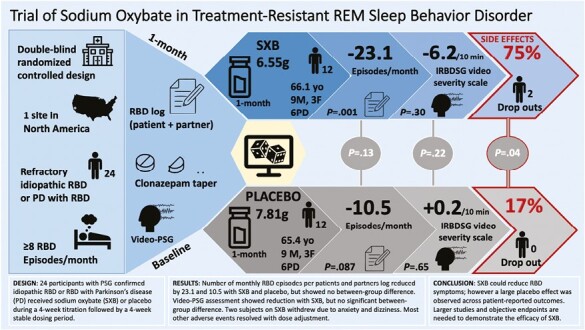

**Statement of Significance**

REM sleep behavior disorder (RBD) is the leading cause of sleep-related injuries but there is currently no symptomatic therapy approved by the Federal Drug Administration. This study is among the rare randomized clinical trials in RBD and the first controlled study using sodium oxybate. This double-blind placebo-controlled study suggests that sodium oxybate may plausibly be effective, not only per patients' report, but per objective video-polysomnography measures. Our findings provide the basis for future larger studies evaluating the efficacy of sodium oxybate in RBD. Importantly, our protocol is the first to implement guidelines from the International RBD Study Group on clinical trials and outcome measures in RBD and use objective endpoints.

## Introduction

Rapid-eye-movement (REM) sleep behavior disorder (RBD) emerges from abnormal motor disinhibition during REM sleep, ranging from small twitches to complex and violent dream-enactment behaviors [1, 2]. Most adult-onset cases are related to the accumulation of alpha-synuclein pathology in the brainstem areas controlling REM sleep, and are thus often associated with Parkinson’s disease (PD) and its long prodromal phase [3]. The unpredictability and potentially lethal risk associated with explosive outbursts of dream enactment significantly affect quality of life for patients and their partners [4, 5]. Clonazepam and melatonin are the mainstays of pharmacotherapy while other drugs have very limited evidence [6, 7].

Sodium oxybate (SXB), a GABA_B_ and GHB receptor agonist, has been used for decades for its sleep-inducing properties [8]. Strong promotion of slow-wave sleep and reduction of disrupted nocturnal sleep could be the primary mechanism for reduction of daytime sleepiness in narcolepsy and other hypersomnias, including that associated with PD [9, 10]. Through modulation of sleep/wake motor control systems, SXB also reduces cataplexy, and possibly RBD behaviors in children with narcolepsy [11]. Several studies have reported the immediate and long-lasting benefit of nightly SXB in treatment-resistant RBD; however, these were anecdotal cases, mostly in isolated RBD (iRBD) [12–15]. Thus, the true efficacy of SXB in RBD remains unknown, including in patients with PD (PD-RBD).

We conducted a randomized, double-blind, placebo-controlled, parallel-group, single-center, phase 2 clinical trial to evaluate the efficacy, and tolerance of nightly SXB in adult patients with iRBD and PD-RBD.

## Methods

### Trial design

This randomized, double-blind, placebo-controlled, parallel-group trial was conducted at the Stanford Sleep Medicine Center. Recruitment started in September 2019 and ended in October 2021 after enrollment of 24 participants and the trial ended in January 2022. Participants were randomly assigned to receive SXB or placebo of similar appearance and taste nightly with 1:1 ratio. The trial consisted of a 4-week baseline, followed by a titration of at least 4 weeks, a 4-week stable dosing period, and a 2-week withdrawal period. All participants provided written consent. The study was approved by the Stanford Institutional Review Board and the design was in accordance with the Declaration of Helsinki. The trial was registered on ClinicalTrials.gov (Identifier: NCT04006925).

### Patients

Patients aged 40 to 85 years with a diagnosis of polysomnography (PSG)-confirmed, definite, iRBD per ICSD-3 criteria [1], or PD and a clinical history of presumed RBD, were recruited from the sleep clinic, via internal and external referrals, and online advertisement. Patients were enrolled (F.Y., A.C., and A.E.) after verification of inclusion and exclusion criteria (E.H.D., M.G.M.). Participants had to fail standard-of-care therapy with clonazepam and melatonin, with persistent RBD symptoms averaging at least two episodes weekly. Treatment failure was defined as either absence of benefit despite adequate doses, intolerance, or contraindication to the drug. A consistent method for monitoring symptoms was required, via a bed partner or home video monitoring device. Exclusion criteria included RBD due to causes other than PD, dementia, congestive heart failure, recent falls or regular use of an assistive device, untreated moderate or severe obstructive sleep apnea based on ≥ 4% decrease in oxygen saturation, and severe restless legs syndrome based on the International Restless Legs Study Group rating scale [16].

### Procedures

After baseline sleep, neurological and psychological assessments, participants entered a 4-week baseline observation period. No change in melatonin or antidepressant treatment was allowed during the study. Participants on clonazepam tapered off this medication during the baseline period to avoid interactions with the study drug. Patients followed a titration schedule of 50% dose reduction weekly until reaching 0.25 mg dose at which point they discontinued the drug. Patients completed an RBD diary daily, after consultation with their bedpartner and/or after review of their home video monitor footages, reporting number, and description of abnormal behaviors. Entry PSG occurred on the last night of the baseline period. Participants using positive airway pressure therapy at home received treatment during PSG. Patients were randomized to SXB or placebo in a 1:1 ratio, using permuted blocks of six, stratified by PD status. Study drug and placebo containers were each labeled with a code assigned by a random number generator. One investigator (CK) was responsible for the random allocation sequence and intervention assignment; all other research personnel and participants were blinded. Starting dose was 4.5 g nightly divided into two doses taken 2.5–4 hours apart, following a prior PD protocol [10]. Efficacy and tolerance were evaluated via weekly phone interviews, and dose was incrementally adjusted until reaching 9 g or the maximum tolerated dose. Per protocol, in patients unable to use two nightly doses due to short sleep times or not tolerating scheduled awakening, single dosing was possible. The dose was fixed during 4 consecutive weeks constituting the treatment period. PSG was repeated on the last night of the treatment period, and participants were reevaluated. During the 2-week observation period, participants could resume prior clonazepam treatment and continued to report RBD episodes.

### Outcome measures

The primary endpoint was the change in number of RBD episodes during treatment versus the baseline period.

Secondary endpoints included overall severity burden, and severe episode burden. Upon review of RBD logs, severity of each episode was rated as mild, moderate, or severe, with respective scores of 1, 5, or 10, replicating the International RBD Study Group (IRBDSG) [17] guidelines for RBD severity scoring based on video polysomnography. The sum of all scores on RBD logs for a given study period was labeled “overall severity burden,” and the number of severe episodes, “severe episode burden.” Other secondary endpoints, per IRBDSG [18], included the clinical global impression (CGI) efficacy scale (CGI-E) jointly filled by patients and partners [19]; CGI improvement scale (CGI-I) per clinician [19]; sleepiness measured by Epworth sleepiness scales [20]. PSGs were scored by sleep technologists and investigators (OSP, AH, MM) blinded to patients’ reported outcomes. PSG measures included: (1) number and severity of RBD motor behaviors per recently issued IRBDSG rules (motor event receiving a weight of 1, 5, or 10 for mild, moderate, and severe), with the only modification that if several motor events were observed during a 30-second epoch of REM sleep, the highest severity score observed was reported. Individual scores were then summed for the entire night (video total movements score), and averaged per 10 minutes of REM sleep [17], (2) total and per 10 minutes of REM sleep number of vocalization in 30-second epochs, and (3) change in REM sleep duration and REM sleep without atonia (RSWA) index measured by two independent sleep experts (O.S.P., A.H.) applying SINBAR criteria using a combination of chin and arm electromyography and 30-second epochs [21]; apnea–hypopnea index; periodic limb movement–arousal index.

Exploratory endpoints were sleep quality, anxiety, mood, and cognition, based on Pittsburgh Sleep Quality Index [22], general anxiety disorder-7 (GAD-7) [23], patient health questionnaire (PHQ-9) [24], and Montreal Cognitive Assessment (MoCA) score [25], respectively.

### Statistical analysis

Statistical analysis (using SAS 9.4, Cary, NC) was by intention-to-treat for the primary efficacy analysis, comparison of baseline characteristics, safety and tolerance, and per-protocol (PP) for secondary/exploratory analyses. PP analysis included all participants who completed titration and the 4-week treatment period. Sample size calculation was based on a prior randomized controlled study by Brunetti et al [26]^.^ To detect an effect size of 50% reduction in the number of RBD episodes per month ([placebo - active]/ placebo) with a power of 80% and alpha of 5%, the number of participants needed to be 10 per arm. Anticipating a dropout rate of 20%, 24 participants were required to be enrolled.

We tested the primary hypothesis that SXB is superior to placebo in reducing the number of RBD episodes from baseline, as observed in the last month of treatment using linear mixed-effects models with random intercepts. Models were parameterized in two ways to estimate separate group trajectories and between-group differences in change over time. Cohen’s d was calculated as a measure of treatment effect size. Similar hypotheses were tested on secondary and exploratory outcomes. Within- and between-group differences were assessed with paired- and unpaired *t*-tests, or with the Wilcoxon Signed Rank and Mann–Whitney U tests for nonnormal data. Participants scoring below 4 on the CGI-I and CGI-E were classified as “responders”; chi-square tests were performed to compare the proportion of responders between groups. All statistical tests were conducted using a two-sided alpha level of 0.05. For secondary/exploratory analyses, bootstrapped percentile-based 95% confidence intervals were calculated based on 10 000 randomly resampled datasets. Descriptive statistics on safety data are provided for all randomized participants (intention-to-treat), by treatment group and study phase.

## Results

**Figure 1.**
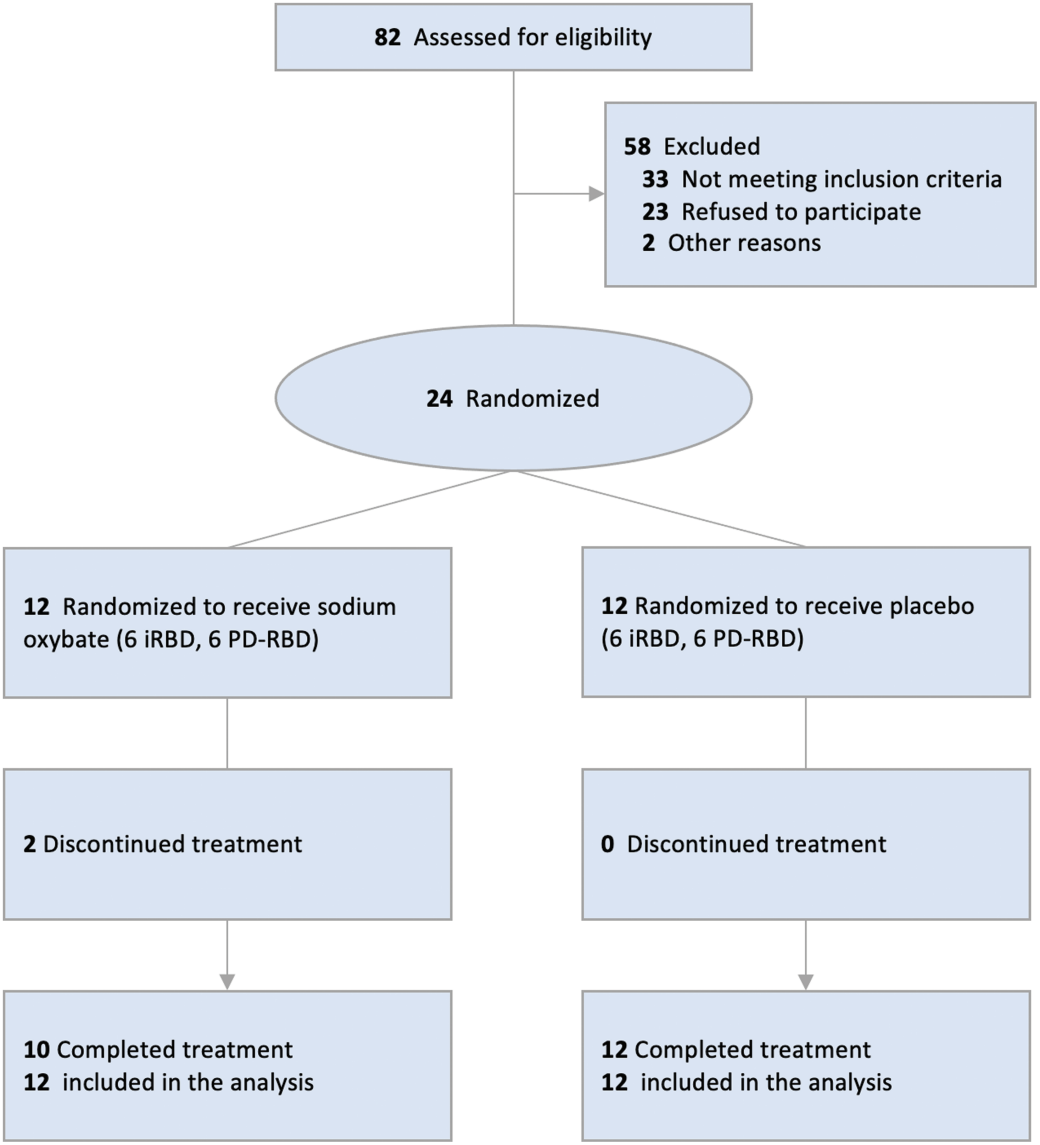
Flow of patients through the trial of sodium oxybate in treatment-resistant rem sleep behavior disorder. Two patients receiving sodium oxybate withdrew from the study during the titration period due to dizziness and severe anxiety. No placebo-treated patients discontinued study drug.

Out of the 82 screened subjects, 58 were excluded: 33 were not eligible due to not meeting inclusion criteria on episode frequency (*n* = 15), unsupported diagnosis (*n* = 8), no prior use of melatonin or clonazepam (*n* = 8) or diagnosis of dementia (*n* = 2), 23 declined participating after discussion of the study protocol, and two for other reasons (travels, coronavirus disease 2019 pandemic) (Figure 1). Twenty-four participants were randomized and received at least one dose of treatment. Definite RBD was confirmed in all participants after PSG review [1]. Of the 24 RBD participants (mean [SD], 65.8 [7.6] years; 18 males [75%], 6 females [25%], 12 iRBD, and 12 PD-RBD), two withdrew due to side effects and 22 completed the study and constitute the PP analysis set. Final mean dose was 6.55 (2.18) g for SXB and 7.81 (2.20) g for placebo. Baseline demographics were similar between treatment groups (Table 1).

**Table 1.** Baseline Characteristics. Intention-to-Treat Analysis in 24 Participants. *N* (%) Unless Stated Otherwise

Characteristic	Treatment group, No. (%)		
	All	Sodium oxybate	Placebo
	*n* = 24	*n* = 12	*n* = 12
*Demographic*			
Age, mean (SD), years	65.8 (7.6)	66.1 (8.2)	65.4 (7.3)
Female	6 (25)	3 (25)	3 (25)
Non-Hispanic White	24 (100)	12 (100)	12 (100)
*Clinical*			
BMI, mean (SD)	25.7 (6.3)	24.4 (4.7)	27.0 (7.6)
RBD symptom duration, mean (SD), years	9.8 (5.8)	11.2 (6.9)	8.4 (4.3)
History of Injuries	22 (92)	10 (83)	12 (100)
Parkinson disease	12 (50)	6 (50)	6 (50)
Sleep apnea	7 (30)	2 (17)	5 (42)
Insomnia	7 (30)	2 (17)	5 (42)
*Current treatment*			
Antidepressant	8 (33)	4 (33)	4 (33)
Clonazepam	9 (38)	5 (42)	4 (33)
Melatonin	14 (58)	8 (67)	6 (50)
Cholinergic	3 (13)	2 (17)	1 (8)
Dopaminergic	10 (42)	6 (50)	4 (33)

BMI, body-mass index; No, number; RBD, rapid-eye-movement sleep behavior disorder; SD, standard deviation.

Both groups included 75% of males, all white, and an equal proportion of iRBD and PD-RBD. Average RBD symptom duration was 9.8 (5.8) years. Twenty-two participants had a history of sleep-related injuries. One-third of participants in each group (*n* = 4) were treated with antidepressants. Most patients were treated with clonazepam (average 0.86 [0.64] mg dose) and/or melatonin (5.54 [6.16] mg dose). Other RBD treatments included transdermal rivastigmine (*n* = 2) and pramipexole (*n* = 1). The SXB and placebo groups were balanced in terms of RBD diagnosis among patients using clonazepam at study entry, with two iRBD and three PD-RBD in the SXB group and two iRBD and two PD-RBD in the placebo group. All participants discontinued clonazepam according to the protocol. Methods for reporting episodes involved a bedpartner in most (*n* = 20) participants, and a video self-monitoring system in six of them. In addition to the two participants who withdrew before completing the study, three could not undergo PSG due to restrictions related to the coronavirus disease 2019 pandemic, three PSGs displayed excessive artifacts due to deep brain stimulation, resulting in only 16 participants (SXB *n* = 7, placebo *n* = 9) with both entry and final PSG data available for PSG analysis.

### Primary outcomes

Groups were statistically equivalent on the primary measure at baseline. Linear mixed models estimated a statistically significant reduction of RBD in the SXB group of 23.1 episodes per month (SE = 6.4, 95% CI −36.0, −10.2, *p* = 0.001, Table 2 model 1) compared to a reduction of 10.5 episodes per month with placebo (SE = 6.0, 95% CI −22.6, 1.6, *p* = 0.08), but the reduction was not significantly different between groups (estimate = -12.6, SE = 8.2, 95% CI −29.2, 4.0, *p* = 0.13, Cohen’s *d* = 0.62) (Table 2, model 2).

**Table 2.** Summary of Parameter Estimates from ITT Linear Mixed Models of Number of RBD Episodes Over Time (Baseline, Titration, Final Dose)

Number of RBD episodes	Estimate (SE)	95% CI	*t*-value	*p*
Model 1				
Intercept	42.8 (4.9)	32.8 to 52.9	8.80	<0.0001
Time × Group = Placebo	−10.5 (6.0)	−22.6 to 1.6	−1.75	0.087
Time × Group = SXB	−23.1 (6.4)	−36.0 to −10.2	−3.62	0.001
Model 2				
Intercept	42.8 (4.9)	32.8 to 52.9	8.80	<0.0001
Time	−10.5 (6.0)	−22.6 to 1.6	−1.75	0.087
Time*Group	−12.6 (8.2)	−29.2 to 4.0	−1.53	0.133

CI, confidence interval; ITT, intention-to-treat; RBD, rapid-eye-movement sleep behavior disorder; SXB, sodium oxybate

Results were similar when within- and between-group changes were analyzed using more conservative nonparametric tests (Table 3).

**Table 3.** Efficacy Measures of RBD Activity Per RBD Logs and Video-Polysomnography

		Median [IQR]							
		Sodium oxybate			Placebo			Sodium oxybate vs. placebo	
		Baseline *n* = 12	Final Dose *n* = 10	Change (95% CI), *p*^a^	Baseline *n* = 12	Final Dose *n* = 12	Change (95% CI), *p*^a^	Difference in Change^b^ (95% CI)	*p* ^c^
**RBD logs**	RBD Episodes per Month	37.5 [27.5 to 48.5]	23.5 [6 to 47]	−23.5 (−41.0 to −2.0), 0.03	35 [28.5 to 45.5]	25.5 [14.5 to 46]	−9.0, (−21.5 to 0.0), 0.11	−14.5 (−32.0 to 11.3)	0.27
	Overall Severity Burden	89.5 [63 to 193.5]	53 [14 to 150]	−46.5 (−85.0 to −19.0), 0.01	121 [78.5 to 149.5]	94.5 [45 to 138]	−34.5 (−52.5 to 1.5), 0.08	−12 (−60.0 to 21.5)	0.46
	Severe Episode Burden	1.5 [0.5 to 4.0]	0.5 [0 to 1]	−1 (−3 to 0), 0.02	1.5 [0 to 3.5]	1.0 [0 to 2.5]	0 (−1 to 1), 0.83	−1 (−3.5 to 0.0)	0.09
		Baseline *n* = 10	Final dose *n* = 7	Change (95% CI), *p*^e^	Baseline *n* = 10	Final dose *n* = 9	Change (95% CI), *p*^e^	Difference in Change^f^ (95% CI)	*p* ^g^
Video-PSG^d^	Video Total Movements	55.5 [18 to 82]	16 [0 to 50]	−25 (−90 to −4), 0.03	57.5 [24 to 269]	25 [16 to 153]	−24 (−61 to 11), 0.15	−1.0 (−82 to 46)	0.72
	Video Movements per 10 minutes	13.2 (12.3)	7.2 (7.4)	−6.2 (−15.2 to 1.7), 0.23	10.1 (8.4)	10.5 (10.0)	0.2 (−4.6 to 4.5), 0.95	−6.4 (−16.2 to 3.1)	0.22
	REM sleep minutes	53.8 (20.1)	31.6 (30.1)	−24.5 (−35.7 to −12.2), 0.01	107.5 (57.2)	81.2 (32.6)	−32.6 (−63.4 to −0.7), 0.09	8.1 (−25.2 to 40.8)	0.67

IQR, interquartile range; PSG, polysomnography.

^a^ *P*-value for within-group change, Wilcoxon Signed Rank test.

^b^negative values favor sodium oxybate group.

^c^ *P*-value for difference between-group comparison, Mann–Whitney U test.

^d^mean (SD) for normal data in both groups.

^e^ *P*-value for within-group change, paired *t*-tests.

^f^Negative values favor sodium oxybate group.

^g^ *P*-value for difference between-group comparison, unpaired *t*-test.

There was no correlation between the reduction in number of RBD episodes and age, gender, or PD status.

### Secondary outcomes

Overall severity burden showed a similar pattern in SXB with 46.5 points median reduction (95% CI −85.0, −19, *p* = 0.01) versus 34.5 points median reduction (95% CI −52.5, 1.5, *p* = 0.08) with placebo, as well as reduction of median monthly severe episodes by 1.0 episode (95% CI −3, 0, *p* = 0.02) versus median increase of 1.0 (95% CI −1, 1, *p* = 0.83) with placebo and no statistically significant difference between groups (*p* = 0.46 for overall severity, *p* = 0.09 for severe episodes) (Table 3). In the SXB group, strong correlation was found between age and reduction in overall severity (Spearman *r* = −0.72, 95% CI −0.92, −0.14, *p* = 0.02) and severe episode burden (Spearman *r* = −0.76, 95% CI −0.94, −0.22, *p* = 0.01), but no correlations with gender or PD status. Responder rate was 83% and 50% with SXB and placebo on CGI-E (*p* = 0.08), and 83% and 58%, respectively on CGI-I (*p* = 0.18). Though not statistically significant, the SXB group showed greater proportion of responders by both measures. Sleepiness was not significantly reduced in either group.

Objective video-PSG scoring demonstrated a significant median reduction of total RBD movements with 25 (95 % CI, −90, 4, *p = 0*.03) point reduction of total movements rating in the SXB group versus 24 (95% CI −61, 11, *p* = 0.15) in placebo, but not per 10 minutes of REM sleep with SXB (Table 3), and no effect on vocalizations. REM sleep was significantly reduced with SXB (*p* = 0.01), but RSWA index did not change. A median reduction of periodic limb movements (PLM)-arousal index was observed with SXB (−2.0, 95% CI, −6.8, 0, *p* = 0.16, *n* = 7) but not with placebo (0.5, 95% CI, 0, 3.5, *p* = 0.13, *n* = 9), between-group difference in change of −0.9 (95% CI, −8.0, −0.1, *p* = 0.04). AHI median reductions were observed in both groups (SXB: −2.0, 95% CI, −17.1, 2.8, *p* = 0.32, *n* = 9; placebo: −3.5, 95% CI, −5.9, −0.2, *p* = 0.01, *n* = 9) but no statistical difference between groups (−0.9, 95% CI, −12.1, 6.5, *p* = 0.66).

### Exploratory outcomes

We found no effect on sleep quality, anxiety, mood, or cognition with either intervention. Unified Parkinson's Disease Rating Scale (UPDRS) and orthostatic vital signs could not be performed due to coronavirus disease 2019-related restrictions.

### Safety analysis

Of the 24 randomized participants who received the study drug, two receiving SXB withdrew due to adverse events (AEs). Average final dose was 6.55 (2.18) g for SXB and 7.81 g (2.20) g for placebo. Two participants, both in the SXB group opted to receive single dose. All-cause treatment-emergent AEs occurred in 11 participants (45.8%): 9 of 12 (75%) in the SXB group, and 2 of the 12 (16.7%) in the placebo group (Table 4).

**Table 4.** Safety Analysis. Number of Participants With an Adverse Event (Percentage Within Study Population) and Total Number of Adverse Events (Percentage of All Adverse Events in the Respective Category)

	Treatment group, No. (%)				
	Sodium oxybate		Placebo		
Adverse events	Participants *n* = 12	Events *n* = 25	Participants *n* = 12	Events *n* = 2	*p*
Any adverse event^‡^	9 (75)	25 (100)	2 (17)	2 (100)	0.004
Not serious	7 (58)	20 (80)	2 (17)	2 (100)	0.037
Serious	5 (42)	5 (20)	0	0	
Unrelated to study medication^†^	1 (8)	1 (4)	0	0	
Potentially related to study medication	9 (75)	24 (96)	2 (17)	2 (100)	0.004
Mild intensity	6 (50)	16 (64)	0	0	
Moderate intensity	3 (25)	5 (25)	2 (17)	2 (100)	0.478
Severe intensity	2 (17)	3 (12)	0	0	
Improvement after dose adjustment or spontaneously	7 (58)	16 (64)	0	0	
Anxiety	1 (8)	1 (6)	0	0	
Asthenia	1 (8)	1 (6)	0	0	
Auditory hallucination	1 (8)	1 (6)	0	0	
Brain fog	2 (17)	2 (12)	0	0	
Enuresis	1 (8)	1 (6)	0	0	
Gastrointestinal discomfort	1 (8)	1 (6)	0	0	
Headache	1 (8)	1 (6)	0	0	
Hot flashes	1 (8)	1 (6)	0	0	
Myalgia	1 (8)	1 (6)	0	0	
Myoclonus episode^*^	1 (8)	1 (6)	0	0	
Nausea	1 (8)	1 (6)	0	0	
Sleep terror^*^	1 (8)	1 (6)	0	0	
Sensation of being “wired”	1 (8)	1 (6)	0	0	
Suicidal ideation^*^	1 (8)	1 (6)	0	0	
Tremor	1 (8)	1 (6)	0	0	
No improvement after dose adjustment	5 (42)	8 (32)	2 (17)	2 (100)	
Anorexia	2 (17)	2 (25)	0	0	
Anxiety^*^	1 (8)	1 (12)	0	0	
Catathrenia	1 (8)	1 (12)	0	0	
Dizziness^*^	1 (8)	1 (12)	0	0	
Hot flashes	0	0	1 (8)	1 (50)	
Increased sweating	2 (17)	2 (25)	0	0	
Insomnia	0	0	1 (8)	1 (50)	
Dysgeusia	1 (8)	1 (12)	0	0	

No, number.

^*^: serious adverse event; ^†^: daytime sedation; ^‡^: Two-sided chi-square or Fisher’s Exact Test testing for group differences in proportion of participants in AE categories where participants are represented in only one of 2 mutually exclusive categories (i.e. serious vs. non-serious AE).

Serious AEs occurred in 5 of the 12 patients (41.7%) with SXB and none with placebo. In the SXB group, other than anxiety and dizziness, which led two participants to withdraw from the study, serious AEs were: myoclonic episodes after first dose only, sleep terror, and suicidal ideation, both of which resolved after dose adjustment. Most common AEs associated with SXB were anorexia, anxiety, increased sweating, and brain fog (*n* = 2, each). Other AEs were sleep-related auditory hallucination, enuresis, catathrenia, daytime sedation, tremor, headaches, hot flashes, dysgeusia, nausea, gastric discomfort, myalgia, and sensation of being “wired” (*n* = 1). AEs in the placebo group were hot flashes (*n* = 1) and insomnia (*n* = 1).

## Discussion

This trial did not demonstrate superiority of SXB over placebo for improving RBD symptoms in treatment-resistant iRBD and PD-RBD. However, SXB resulted in significant improvements across all subjective outcomes of RBD activity, which was not seen with placebo. Eight out of ten patients receiving SXB had reductions in episode frequency above the placebo mean of 15%. Subjective response was supported by objective reduction of RBD movements observed on video-PSG. Taken together, these findings suggest that SXB may be an effective therapy for severe, treatment-resistant RBD, but due to an inconsistent response and a large placebo effect, statistical significance was not reached.

Two participants (17%) receiving SXB could not tolerate side effects, another two reported no benefit or paradoxical worsening despite receiving a 9 g dose. The latter two patients were both in their 50 seconds and had a diagnosis of iRBD. Correlation analysis shows that younger age was associated with worse treatment responses. It is also possible that those participants’ RBD phenotype differed from the rest of the group. As biomarkers of phenotypic progression in synucleinopathy are being developed [27], including them in future drug trials could inform potential factors influencing treatment response.

There is currently no validated method to objectively monitor outcomes in RBD [28]. Other reports of video-PSG outcomes with SXB in iRBD [14] showed no effect; however, they used a different scoring method, only reporting severity, not number of episodes [29]. We applied the IRBDSG video scoring rules to measure RBD activity before and after treatment [17]. RBD behaviors have an important night-to-night variability [29, 30]. Remarkably, despite a small sample size (*n* = 16), significant effect was found only with SXB. The same measure averaged per 10 minutes of REM sleep showed no effect, possibly due to REM sleep suppression with SXB. This would support the notion that observed improvements in RBD movements may predominantly be due to REM sleep suppression.

Little is known about the mechanism of action of SXB in RBD. Given its short half-life and duration of action, SXB is usually recommended to be taken twice per night, however, we opted in this study to have the flexibility of single dosing for promoting patient adherence and for exploring differential effect compared to double dosing. The literature on efficacy in RBD is limited to six patients with iRBD and one with PD-RBD [12–15]. Limited data suggest that SXB may selectively reduce most injurious and complex dream-enactment episodes [14]. In our dataset, mild episode severity did not predict treatment failure. Contrasting with RSWA index reduction observed in narcolepsy patients [11, 31], we found, like others, no such reduction with SXB [14]; however, REM sleep duration reduced by 52% (−24.5 [17.5] minutes, *p* = 0.01) with complete suppression of REM sleep in two patients treated with SXB. SXB promotes slow-wave sleep, reduces arousal, and suppresses REM sleep. Conceivably, these three mechanisms could contribute to its efficacy in RBD. Among these three mechanisms, REM sleep suppression may be a primary mechanism accounting for the overall reduction of RBD burden with SXB since no effect was found when motor events were reported per 10 minutes of REM sleep. Until further research is conducted on the effects of REM sleep suppression in patients with prodromal or manifest PD, suppression of REM sleep shall not be the sole mechanism accounting for a reduction of RBD symptoms.

AEs occurred in 75% and serious AEs in 42% of patients receiving SXB, leading two participants to discontinue therapy. As expected, non-serious AEs were more frequent (58%) with SXB than placebo (17%). Neither PD nor age correlated with frequency or severity of AEs. This low tolerability contrasts with prior studies in PD [10], but was observed in a recent trial despite lower starting dose of 3 g [9]. Time-locked prolonged myoclonus after SXB intake was unexpected but resolved without reappearing during uptitration to 8 g. In future studies, lower starting doses and slower titration may be considered. Altogether, limited tolerance for a significant portion of patients with iRBD or PD could limit the use of SXB, and warrants systematic monitoring of neuropsychiatric complications.

Limitations of this study include the small sample size and large variability in the data. The large benefit observed in the placebo arm was not expected. A new sample size calculation reveals that 65 patients per arm would be required to detect statistical differences between interventions using a parallel arm design, and that 38 patients would be required in a crossover design. Prior RCTs monitoring episode frequency reported a similar placebo effect [32], which may be reduced in those using a crossover design [25, 33]. Crossover designs may attenuate placebo effect since they require participants to compare efficacy between interventions which may allow them to be biased by their own assumptions about the treatment sequence. In our study, bedpartners were generally the only source for reporting outcomes as patients were mostly unaware of dream enactments. Conceivably, bedpartners’ own expectations may reduce anxiety and improve their sleep, which might lead to an underreporting of RBD behaviors and an amplification of placebo effect by proxy. Future studies may consider using home video monitoring for objective measures of symptomatic response.

Other possible limitations are related to the difficulty to control for confounding effects of clonazepam and melatonin. The study design gave careful consideration to safety and efficacy parameters. Discontinuing all treatments in participants with uncontrolled RBD, as it can lead to severe exacerbation of RBD symptoms and injuries to patients and their bed partners, was not a viable option. However, the concomitant use of clonazepam and SXB can potentially lead to central nervous depression. To maintain adequate level of safety for patients and their partners and adequately evaluate SXB, patients were required to reduce clonazepam treatment by tapering down to the lowest possible dose and discontinue, when possible, while any non-sedative RBD treatment would be maintained during the study, including melatonin. The majority of participants in this study were treated with melatonin, with no difference between groups. There are no known pharmacological interactions between melatonin and SXB, thus low concern about a potential bias.

The strengths of this study are that it is the first double-blind, placebo-controlled study to systematically evaluate the effects of SXB on RBD, the individualized titration scheme, use of RBD logs not just reporting episode frequency but informing on their severity, and objective measure of RBD activity per video-PSG.

Our results suggest that although SXB could plausibly be an effective therapy in severe RBD, heterogeneous and unpredictable responses may limit its use until more research is conducted. A significant within-group effect was observed on objective RBD activity per video-PSG. To the best of our knowledge, the present study is the second one implementing recommendations from the IRBDSG on video analysis for measuring outcomes in RBD [34]. This approach should be considered for systematic inclusion in future trials. Studies should also explore the utility of smart devices, such as home video monitoring and wearables for monitoring treatment outcomes. Future studies with larger patient samples and consideration of crossover design are needed to determine individual factors, and possibly RBD phenotypes that could influence treatment outcomes and tolerability with SXB. Until then, this drug may remain a potentially effective therapy that requires close monitoring of treatment-emergent AEs.

## Data Availability

The data underlying this article will be shared on reasonable request to the corresponding author.
