# Correction to: Sodium oxybate in treatment-resistant rapid-eye-movement sleep behavior disorder

**DOI:** 10.1093/sleep/zsad149

**Published:** 2023-05-26

This is a correction to: Emmanuel H During and others, Sodium oxybate in treatment-resistant rapid-eye-movement sleep behavior disorder, *Sleep*, 2023;, zsad103, https://doi.org/10.1093/sleep/zsad103

In the originally published version of this manuscript, the following funding statement was erroneously omitted:

“This work was supported by Jazz pharmaceuticals.”

This error has now been corrected.

